# Using Twitter to investigate opinions about multiple sclerosis treatments: a descriptive, exploratory study

**DOI:** 10.12688/f1000research.5263.1

**Published:** 2014-09-10

**Authors:** Sreeram Ramagopalan, Radek Wasiak, Andrew P. Cox

**Affiliations:** 1 Evidera, London, W6 8DL, UK

## Abstract

**Background:** Multiple sclerosis (MS) is a common complex disorder, with new treatment options emerging each year. Social media is being increasingly used to investigate opinions about drugs, diseases and procedures. In this descriptive exploratory study, we sought to investigate opinions about currently available MS treatments.

**Methods:** The Twitter resource Topsy was searched for tweets mentioning the following MS treatments: Aubagio, Avonex, Betaferon or Betaseron, Copaxone, Extavia, Gilenya, Lemtrada, Novantrone, Rebif, Tysabri and Tecfidera between 1 Jan 2006 to 31 Jul 2014. Tweets were normalised and sentiment analysis performed.

**Results: **In total, there were 60037 unique tweets mentioning an MS treatment. About half of the tweets contained non-neutral sentiment. Mean sentiment scores were different for treatments ranging from -0.191to 0.282 when investigating all tweets. These differences in sentiment scores between treatments were statistically significant (P<0.001). Sentiment scores tended to be higher for oral MS treatments than injectable treatments.

**Conclusions:** Many tweets about MS treatments have a non-neutral sentiment. The analysis of social media appears to be a potential avenue for exploring patient opinion about MS treatments.

## Introduction

The analysis of social media is becoming a powerful tool that is being used increasingly to answer research questions across numerous areas including disease spatio-temporal epidemiology and drug adverse events ^1– 3^. Key stake-holders in the pharmaceutical industry, including patients, physicians, regulatory authorities and pharmaceutical companies, are increasingly using web technologies such as social media, blogs and forums to generate and access opinions and real-world evidence of potentially medically important issues. This content serves as an important source of on-line medical opinions, information and sentiments relating to particular drugs and events. The underlying assumption is that with access to such information, a patient will be able to make more informed decisions about drugs, diseases, procedures and health-care providers.

Multiple sclerosis (MS) is a chronic, neurodegenerative autoimmune disorder of the central nervous system (CNS). With a prevalence of one per 800 in North America and Northern Europe, MS is the most common acquired neurological disorder in young adults ^4^. About 85% of patients present initially with relapsing-remitting MS (RRMS), characterized by recurrent episodes of neurological dysfunction interspersed with periods of lack of apparent disease activity ^4^.

At present, there are nine disease modifying therapies (DMTs) approved by the US Food and Drug Administration (FDA) and 10 DMTs approved by the European Medicines Agency (EMA) for the treatment of RRMS, with new treatment options emerging each year. Approved treatments include interferons (Avonex, Betaferon, Betaseron, Extavia, Rebif), glatiramer acetate (Copaxone), natalizumab (Tysabri), and, more recently, the oral treatments teriflunomide (Aubagio), fingolimod (Gilenya), and dimethyl fumarate (Tecfidera). In this study, we explored whether we could analyse social media to help gauge patient sentiment about treatments using MS as an example. We used the popular social media site Twitter ( http://twitter.com) to explore the reporting of patient sentiment and emotions about MS treatments.

## Methods

### Data

The Twitter resource, Topsy ( http://topsy.com/), which houses all tweets made since 2006, was searched for the following brand names of MS treatments: Aubagio, Avonex, Betaferon or Betaseron, Copaxone, Extavia, Gilenya, Lemtrada, Novantrone, Rebif, Tysabri and Tecfidera using a daily search-time window (i.e. searching for tweets made every day), and specifying the English language. Brand names were used as we thought this would be more likely to reflect patient tweets and further the generic name for some MS treatments are not specific MS treatments. All dates from 1 Jan 2006 to 31 Jul 2014 were searched. For days in which there were more than 1000 tweets satisfying the search criteria, an hourly search-time window was applied for that day, to enable all available tweets to be found (the resource limits searches to 1000 results).

Tweets were downloaded in Extensible Markup Language (XML) format from topsy.com using the application program interface, otterapi ( https://code.google.com/p/otterapi/).

### Data filtering

Tweets were subsequently filtered to generate datasets for analysis:

1. A unique dataset was generated from the “highlight” data class; thus, removing all directly copied retweets. This was performed so that sentiment analysis could be performed on unique tweets and not bias analyses by having several copies of the same tweet.

All subsequent filtering was case-insensitive.

2. The unique dataset from (1) was filtered to remove items relating to company share prices/stockmarket news.

This was achieved by removing all tweets that contained:

a) “market_jp”, “thestreet”, “rtebusiness”, “pharma”,or “pharmsales” in the “permalink” dataclass,

or

b) “bloomberg”, “forbes”, “dow jones”, “financial times”, “stockpickr”, “marketwatch”, “business:”, “profit”, “shares”, or “sec” in the “highlight” data class. This filter was performed as we wanted to identify patient opinion about MS treatments and not stock market related tweets. This filter did retain tweets containing company names, some of which were stock/share price related but some tweets containing company names were from patients.

3. The dataset from (2) was further filtered to remove items that mentioned the manufacturing companies by name: tweets were removed if they contained any of the following:

“novartis”, “elan”, “biogen”, “merck”, “bayer”, “genzyme”, “sanofi”, “teva”, or “serono”. This filter was stringent and removed the majority of stock/share related tweets, but also removed some patient tweets.

### Normalisation

Because of the short nature of tweets, typographical errors, ad-hoc abbreviations, phonetic substitutions, ungrammatical structures and emoticons are common, causing problems for text processing tools. Tokenisation and normalisation to make better sense of the tweet texts was achieved using TwitIE ( http://gate.ac.uk/sale/ranlp2013/twitie/twitie-ranlp2013.pdf?m=1). Normalisation did not remove or alter any of the drug names.

### Sentiment and word frequency analysis

Tweets were grouped into sequential monthly time periods for sentiment analysis using the twitteR R package ( https://github.com/geoffjentry/twitteR/) and Jeffrey Breen’s sentiment analysis code ( https://github.com/jeffreybreen/twitter-sentiment-analysis-tutorial-201107; a tutorial can be found at: http://www.inside-r.org/howto/mining-twitter-airline-consumer-sentiment). Word frequency analysis in tweets was performed using TagCrowd ( http://tagcrowd.com). TagCrowd uses language-specific lists of common words which are removed from analysis.

### Statistical analysis

Using lists of 2006 positive and 4783 negative words ( http://www.cs.uic.edu/~liub/FBS/sentiment-analysis.html#lexicon), the sentiment score for any tweet is calculated as follows:

*Sentiment score = number of positive words - number of negative words*

If the sentiment score > 0, this means that the sentence has an overall 'positive opinion', if the sentiment score < 0, this means that the sentence has an overall 'negative opinion', if the sentiment score=0, then the sentence is considered to be a 'neutral opinion'. Sentiment scores were summed for all tweets for each MS treatment, and means calculated. Mean sentiment scores were compared across treatments using the Kruskal-Wallis test. Statistical analysis was performed using R version 3.1.1 and p values less 0.05 were considered significant.

## Results

**Figure 1.**
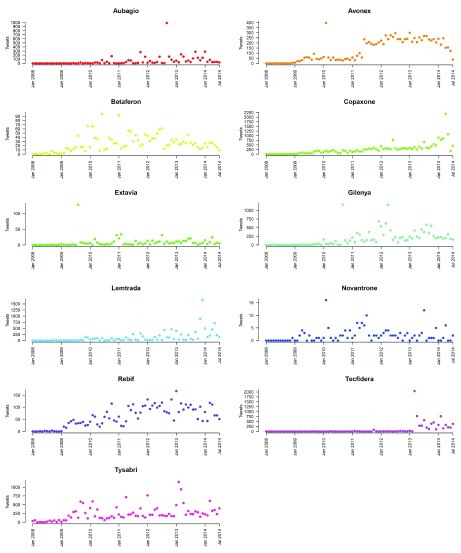
Number of tweets by month for each treatment.

In total, there were 60037 unique tweets mentioning an MS treatment. The number of tweets by month is shown in Figure 1. Tweets for Tysabri started the earliest (January 2008) and Aubagio the latest (February 2009). When removing tweets that included share/stock information there were 56708 unique tweets and when removing tweets that included share/stock information or company names there were 41690 unique tweets.

The number of tweets by treatment, overall and when removing tweets that included share/stock information and/or company names is shown in Table 1. Tysabri had the largest number of tweets (n=14542, all tweets; n=10984 after filtering for company names and stock/share tweets) and Novantrone had the lowest (n=110, all tweets; n=109 after filtering for company names and stock/share tweets), both before and after filtering.

**Table 1.** Number of tweets by treatment.

Treatment	Aubagio	Avonex	Betaferon	Copaxone	Extavia	Gilenya	Lemtrada	Novantrone	Rebif	Tecfidera	Tysabri
**First Tweet Month**	Feb 2009	Apr 2008	May 2008	Apr 2008	Sep 2008	Apr 2008	Oct 2008	Dec 2008	Apr 2008	Oct 2008	Jan 2008
**Total Number of Tweets**	2814	4643	1615	11634	511	9376	5634	110	4236	4922	14542
**Total Number of Tweets with** **Stock Tweets Removed**	2632	4540	1577	10676	501	8902	5302	110	4162	4637	13669
**Total Number of Tweets with** **Stock and Company Names** **Removed**	1601	4214	1392	6818	432	6999	2444	109	3784	2913	10984

The sentiment score analysis of all normalised tweets, normalised tweets excluding those that contained share/stock information and normalised tweets excluding those that contained share/stock information and company names are shown in Table 2, Table 3 and Table 4. About half of all tweets in all analyses had a neutral sentiment (43–61%, all tweet data; 45–57% after filtering for company names and stock/share tweet data). Tweets for drugs that contained sentiment were more likely to be positive sentiment, apart from tweets for Novantrone and Tysabri (23–33% for drugs apart from Novantrone (16%), all tweet data; 24–31% for drugs apart from Novantrone (17%) and Tysabri (28%), after filtering for company names and stock/share tweet data).

**Table 2.** Sentiment analysis of all normalised tweets.

Treatment	Aubagio	Avonex	Betaferon	Copaxone	Extavia	Gilenya	Lemtrada	Novantrone	Rebif	Tecfidera	Tysabri
**Proportion of Tweets with** **Positive Sentiment**	0.303	0.277	0.241	0.293	0.233	0.318	0.299	0.164	0.265	0.33	0.323
**Proportion of Tweets with** **Negative Sentiment**	0.118	0.224	0.218	0.216	0.162	0.155	0.18	0.3	0.248	0.123	0.252
**Proportion of Tweets with** **No Sentiment**	0.579	0.499	0.541	0.491	0.605	0.527	0.521	0.536	0.487	0.548	0.425
**Summed Sentiment Score**	777	263	32	1081	25	2111	920	-21	74	1388	1513
**Mean Sentiment Score** **(Standard Deviation)**	0.276 (0.91)	0.057 (1.095)	0.02 (0.984)	0.093 (1.031)	0.049 (0.854)	0.225 (0.949)	0.163 (0.959)	-0.191 (1.054)	0.017 (1.101)	0.282 (0.932)	0.104 (1.149)

**Table 3.** Sentiment analysis of all normalised tweets with tweets containing share/stock information excluded.

Treatment	Aubagio	Avonex	Betaferon	Copaxone	Extavia	Gilenya	Lemtrada	Novantrone	Rebif	Tecfidera	Tysabri
**Proportion of Tweets with** **Positive Sentiment**	0.299	0.275	0.236	0.293	0.236	0.315	0.296	0.164	0.267	0.328	0.319
**Proportion of Tweets with** **Negative Sentiment**	0.119	0.227	0.221	0.214	0.162	0.156	0.183	0.3	0.25	0.123	0.252
**Proportion of Tweets with** **No Sentiment**	0.582	0.498	0.543	0.493	0.603	0.53	0.521	0.536	0.483	0.549	0.43
**Summed Sentiment Score**	712	231	4	1091	26	1977	818	-21	69	1313	1278
**Mean Sentiment Score** **(Standard Deviation)**	0.271 (0.911)	0.051 (1.099)	0.003 (0.973)	0.102 (1.035)	0.052 (0.859)	0.222 (0.951)	0.154 (0.962)	-0.191 (1.054)	0.017 (1.107)	0.283 (0.934)	0.093 (1.149)

**Table 4.** Sentiment analysis of all normalised tweets with tweets containing share/stock information and company names excluded.

Treatment	Aubagio	Avonex	Betaferon	Copaxone	Extavia	Gilenya	Lemtrada	Novantrone	Rebif	Tecfidera	Tysabri
**Proportion of Tweets with** **Positive Sentiment**	0.299	0.264	0.239	0.29	0.255	0.284	0.311	0.165	0.267	0.283	0.275
**Proportion of Tweets with** **Negative Sentiment**	0.128	0.237	0.224	0.221	0.185	0.164	0.195	0.303	0.26	0.143	0.277
**Proportion of Tweets with** **No Sentiment**	0.573	0.499	0.537	0.489	0.56	0.553	0.493	0.532	0.473	0.574	0.448
**Summed Sentiment Score**	395	120	4	677	20	1149	378	-21	12	523	-115
**Mean Sentiment Score** **(Standard Deviation)**	0.247 (0.923)	0.028 (1.11)	0.003 (0.999)	0.099 (1.077)	0.046 (0.91)	0.164 (0.933)	0.155 (1.034)	-0.193 (1.058)	0.003 (1.136)	0.18 (0.914)	-0.01 (1.129)

**Figure 2.**
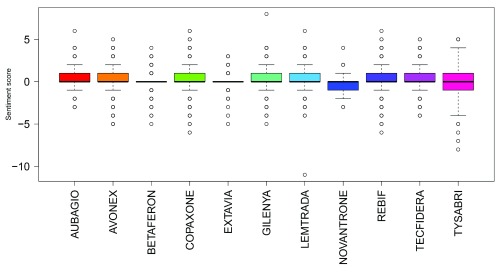
Boxplots of sentiment scores for all normalised tweets.

**Figure 3.**
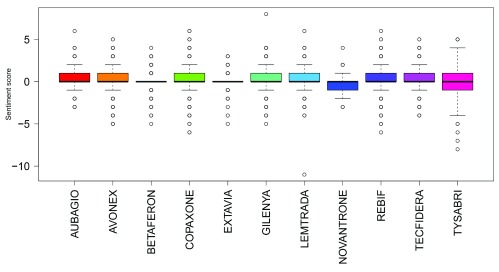
Boxplots of sentiment scores of all normalised tweets with tweets containing share/stock information excluded.

**Figure 4.**
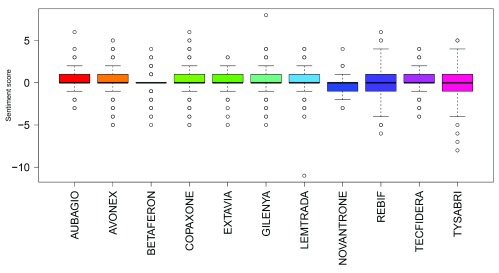
Boxplots of sentiment scores of all normalised tweets with tweets containing share/stock information and company names excluded.

Summing sentiment scores for all tweets showed positive overall sentiment scores for all drugs apart from Novantrone (all analyses) and Tysabri (only after filtering for company names and stock/share tweet data). Gilenya had the highest summed sentiment score in all analyses. Boxplots of sentiment scores of all normalised tweets, normalised tweets excluding those that contained share/stock information and normalised tweets excluding those that contained share/stock information and company names are shown in Figure 2, Figure 3 and Figure 4. The mean sentiment score ranged from -0.191 to 0.282 (all tweet data); and -0.193 to 0.247 (after filtering for company names and stock/share tweet data). Novantrone always had the lowest mean sentiment score. Tecfidera had the highest mean score in the all tweet data, and Aubagio had the highest mean score in the filtered for company names and stock/share tweet data. The mean sentiment scores were different in all analyses (P<0.001 in the all tweet data, filtered for stock/share tweet data and filtered for company names and stock/share tweet data).

**Figure 5.**
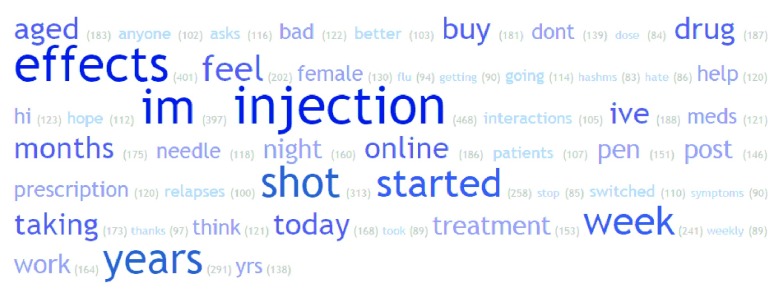
Word cloud for all normalised tweets for Avonex.

**Figure 6.**
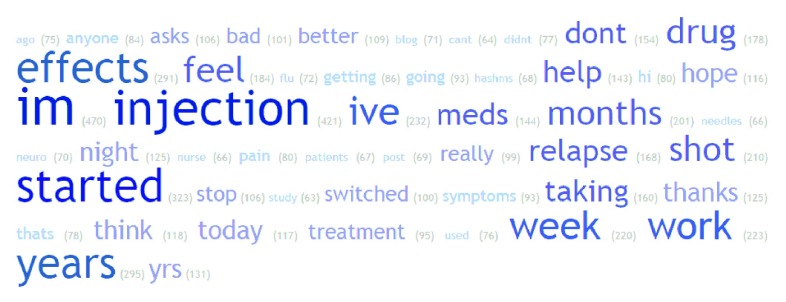
Word cloud for all normalised tweets for Rebif.

**Figure 7.**
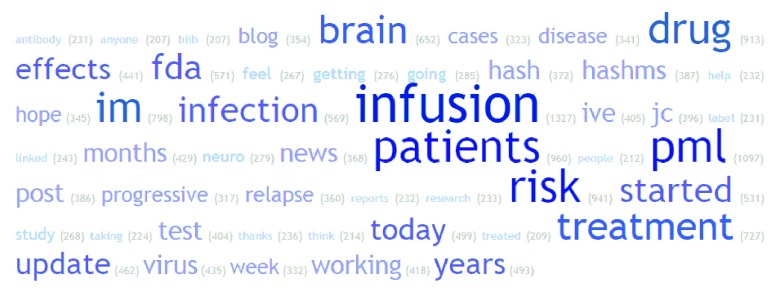
Word cloud for all normalised tweets for Tysabri.

Most common words in tweets for treatments were investigated. Example word clouds for the 50 most common words (excluding commonly used English words and drug names) in all normalised tweets for Avonex, Rebif and Tysabri are shown in Figure 5, Figure 6 and Figure 7. Of note is the frequency of ‘flu’ and ‘injection’ in Avonex and Rebif tweets and ‘infusion’ and ‘pml’ in Tysabri tweets.

## Discussion

We present here, to the best of our knowledge, the first analysis of social media for MS treatments. A significant proportion of tweets did contain non-neutral sentiment about MS treatments, and the distribution of sentiment score was different between treatments. Thus it appears that Twitter can be a potential resource to understand patient opinion about MS treatments. When looking at frequency of words, notably ‘flu’ and ‘injection’ were in the 50 most common words in tweets about Rebif and Avonex and ‘infusion’ and ‘pml’ in the 50 most common words in tweets about Tysabri. Flu-like symptoms are well known side-effects of the injectible treatments Rebif and Avonex and progressive multifocal leukoencephalopathy or ‘pml’, is a well-known risk for patients taking the intravenously infused Tysabri ^5^. This provides some sort of face validity for our results reflecting real specific tweets about MS treatments.

Interestingly, the oral MS treatments- Gilenya, Aubagio and Tecfidera had the highest mean sentiment scores and Gilenya had the highest summed sentiment score in all analysis. This may reflect a well known patient preference for oral therapies as compared to injectible treatments ^6, 7^. Further work is needed to explore tweets in detail to see if the higher mean sentiment scores are related to positive tweets about the fact that these drugs are to be taken orally.

There are a number of limitations to this study. We are using automated tools to assign sentiment to tweet content- these tools will not recognise the intricacies of human language e.g. the context of the tweet and sarcasm for example. Further, whilst we tried to normalise tweets, the diversity of twitter slang will mean that abbreviations may not be recognised. We may have underestimated the number of tweets as we used brand names to identify drugs. Any tweets using the generic name or shortened versions will be missed. Whilst we tried to focus on tweets from patients, it is inevitable that business related tweets will have been included in our analysis and some patient tweets lost during filtering. It is also possible that not all tweets were delivered to us by the Twitter interface, although that is not possible to verify.

Our findings and any interpretation should be regarded as speculative and exploratory. The results represent what can be potentially done relatively quickly and easily using data from Twitter. More rigorous analytical methods can be applied for more specific questions (e.g. the analysis of adverse events). It is clear from this study that tweets are written about MS treatments and many of these have a non-neutral sentiment. Further work is needed to look at these tweets in detail to further understand patient opinion about MS treatments.
