# Supplementary material for: Using Twitter to investigate opinions about multiple sclerosis treatments: a descriptive, exploratory study
Source: F1000Res. 2014 Sep 10;3:216. [Version 1] doi: 10.12688/f1000research.5263.1 (PMC4264640; doi:10.12688/f1000research.5263.1)
Supplement: Peer review history

# Peer review: Referee response for version 1

**DOI:** 10.5256/f1000research.5610.r6809

**Published:** 2014-12-09

**Authors:** Michael Hutchinson

**Affiliations:** 1 St Vincent's University Hospital, University College Dublin, Dublin, Ireland

An interesting paper and an introduction to the reviewer of the new methods of analysis of social media. The authors have emphasised the pains that they took to exclude tweets from company and financial sources. The difficulty is that one cannot be certain that what they are analysing is not contaminated by tweets stimulated in some way from particular pharmaceutical companies. There is, of course, a "newness" affect and this explains the frequency of the tweets for Aubagio and Tecfidera. If one excludes Novantrone and Tysabri, then the lowest sentiment scores are for the interferons. What I find remarkable in this report is that the highest mean sentiment score is for Aubagio, a drug which many neurologists would have less enthusiasm for, given problems in its use in patients of childbearing age. Interesting findings and nicely presented paper, but I am still concerned about the source data; are all these tweets from genuine patients?

I have read this submission. I believe that I have an appropriate level of expertise to confirm that it is of an acceptable scientific standard.

# Peer review: Referee response for version 1

**DOI:** 10.5256/f1000research.5610.r6808

**Published:** 2014-11-28

**Authors:** Bruce Taylor

**Affiliations:** 1 Menzies Research Institute Tasmania, University of Tasmania, Hobart, Australia

I found this a very interesting report and feel that as the authors do that this method of analysis may provide a real world window into patient perceptions of treatment, efficacy, and side effects. It would be interesting to assess churn in the twitter responses, for example PML in Tysabri is a major issue to patients and it would be interesting to see how this has trended over time, as the period for which tweets were collected includes the period of time when PML was a major issue (newly described) and would have been a major source of negative sentiment. Similarly for the newer agents it would be interesting to track these findings over time from release date and see whether positivity or negativity changes with patient experience.

This type of research will undoubtedly provide significant and important patient focused outcomes for future medication usage and tolerance studies. at considerable cost efficiency.

Can the authors look at trending words in these tweets, for example PML in Tecfidera or hair loss for Aubagio?

I have read this submission. I believe that I have an appropriate level of expertise to confirm that it is of an acceptable scientific standard.
